# Shifting paradigms in the approach to footstrikes, footwear and treatment of the foot

**DOI:** 10.1186/1757-1146-4-S1-A3

**Published:** 2011-05-20

**Authors:** Irene Davis

**Affiliations:** 1 Dept. of Physical Medicine and Rehabilitation, Harvard Medical School, Cambridge, MA, 02138, USA

## 

The recent rise in popularity of barefoot and minimal footwear running has led to spirited debates within the scientific, clinical and public arenas. While there are no definitive injury studies that support barefoot over shod running, there is a growing body of literature that suggests we may need to change our thinking about running mechanics, footwear and how we treat the foot. This presentation will review existing studies of the relationship between shod footstrikes, mechanics and injury. In addition, the direct effect of footwear on mechanics will be discussed. Finally, this evidence will also be used to suggest a change in our approach to common foot related pathologies. It is hoped that this presentation will be a catalyst for changing the way we view these issues.

